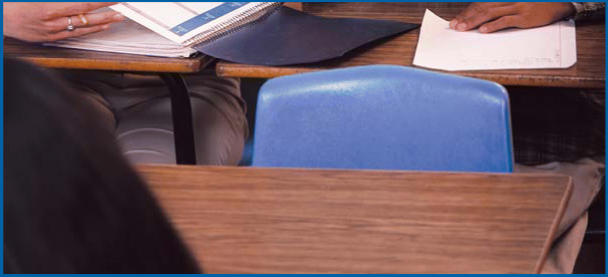# Headliners: Environmental Tobacco Smoke: Respiratory Effects Linked to Genetic Susceptibility

**Published:** 2006-04

**Authors:** Tanya Tillett

Wenten M, Berhane K, Rappaport EB, Avol E, Tsai W-W, Gauderman WJ, et al. 2005. *TNF*-308 Modifies the Effect of Second-Hand Smoke on Respiratory Illness–Related School Absences. Am J Respir Crit Care Med 172:1563–1568.

Children are at special risk for adverse effects from exposure to secondhand smoke (SHS). Estimated population-attributable risks for SHS exposures in children range from 9% for asthma prevalence to 25% for hospital admissions due to lower respiratory symptoms. According to the Third National Health and Nutrition Examination Survey, 43% of children between the ages of 4 and 11 years are exposed to SHS at home. Now NIEHS grantees Frank D. Gilliland, Rob McConnell, W. James Gauderman, Louis Dubeau, Edward Avol, and Kiros Berhane, with their colleagues at the University of Southern California Keck School of Medicine, have shown that children with a particular genetic makeup are at a substantially greater risk for respiratory illness when exposed to SHS.

Using data from the Children’s Health Study, the team examined school absences for 1,351 fourth grade students from 27 California elementary schools between January and June 1996. They categorized illness-related absences as being due to nonrespiratory or respiratory illness, then divided the latter into upper respiratory illness (runny nose/sneezing, sore throat, earache) or lower respiratory (wet cough, wheeze, asthma). They also gathered information on the students’ health history, including history of asthma, and their exposure to smoking and allergens at home.

The researchers also collected buccal cells from each subject, to determine the student’s tumor necrosis factor (TNF)–α genotype. TNF-α is an important cytokine in the inflammatory response to SHS. The *TNF* gene has a common variant in the promoter region G-308A that has been associated with TNF-α expression regulation in some studies.

Students who were exposed to SHS at home had a 51% greater risk of having a lower respiratory illness–related school absence compared with unexposed students. The association was clearest in students who had at least one copy of the variant A allele on *TNF*-308. Students who displayed the AA or AG genotype had a 75% increase in risk of illness-related absences of any kind. Those children possessing the A variant who were exposed to SHS at home had an even more pronounced risk for respiratory illness–related absences, especially absences due to lower respiratory illness. When compared to nonexposed children with the GG genotype, children with the A allele who were exposed to two or more smokers in the home were four times as likely to stay home because of lower respiratory illness.

The researchers postulate that variations in the *TNF* gene might intensify the body’s inflammatory response to oxidative stress caused by cigarette smoke. They also note that since a significant number of people are exposed to SHS, future studies should focus on identifying genetically susceptible groups so actions can be taken to reduce their exposure.

## Figures and Tables

**Figure:**